# Alternating Current Electrophoretic Deposition of Antibacterial Bioactive Glass-Chitosan Composite Coatings

**DOI:** 10.3390/ijms150712231

**Published:** 2014-07-09

**Authors:** Sigrid Seuss, Maja Lehmann, Aldo R. Boccaccini

**Affiliations:** Institute of Biomaterials, University of Erlangen-Nuremberg, Cauerstrasse 6, Erlangen 91058, Germany; E-Mails: sigrid.seuss@fau.de (S.S.); maja.lehmann@kabelmail.de (M.L.)

**Keywords:** electrophoretic deposition, chitosan, bioactive glass, antibacterial, coatings

## Abstract

Alternating current (AC) electrophoretic deposition (EPD) was used to produce multifunctional composite coatings combining bioactive glass (BG) particles and chitosan. BG particles of two different sizes were used, *i.e.*, 2 μm and 20–80 nm in average diameter. The parameter optimization and characterization of the coatings was conducted by visual inspection and by adhesion strength tests. The optimized coatings were investigated in terms of their hydroxyapatite (HA) forming ability in simulated body fluid (SBF) for up to 21 days. Fourier transform infrared (FTIR) spectroscopy results showed the successful HA formation on the coatings after 21 days. The first investigations were conducted on planar stainless steel sheets. In addition, scaffolds made from a TiAl4V6 alloy were considered to show the feasibility of coating of three dimensional structures by EPD. Because both BG and chitosan are antibacterial materials, the antibacterial properties of the as-produced coatings were investigated using *E. coli* bacteria cells. It was shown that the BG particle size has a strong influence on the antibacterial properties of the coatings.

## 1. Introduction

Electrophoretic deposition (EPD) is a two-step process for depositing coatings from suspended particles and polymer molecules [1,2,3]. EPD requires the homogeneous dispersion of charged particles in a suspension medium. The suspension should be stable over the deposition period to prevent uncontrolled microstructure of the coatings. In the standard EPD process, two electrodes—the working electrode (substrate material) and the counter electrode—are immersed in the suspension. When applying an electric field, the particles move towards the oppositely charged substrate material, where in the next step they deposit to form a coating. The advantages of EPD are the rather simple equipment used, the flexibility in substrate shape/dimension and coating material choice as well as the ability to easily control the homogeneity and thickness of the coatings [2]. Concerning the deposition material, it is necessary that the coating material is available as a powder or colloid. The range of materials that can be used for EPD is wide. In early studies, EPD was used mainly in traditional ceramic coating processing, but nowadays, the field of applications and materials is broad including a wide range of nanomaterials, polymers and composites [4]. In recent years, EPD has been gaining increasing interest as a processing method for biomaterials, in particular biomedical coatings, including ceramic and polymeric materials and their composites [2]. In addition, EPD of bacterial cells, enzymes or proteins is being increasingly investigated [5]. In most cases, a direct current (DC) is used for the deposition process. However, when using aqueous suspensions, application of DC can lead to undesired electrolysis of water at the electrodes. If this effect occurs, bubbles can be entrapped in the coatings, reducing the coating quality and adhesion to the substrate. This is the reason why increasing attention is being paid to EPD under alternating current (AC) conditions [6]. AC–EPD reduces bubble formation at the electrode due to the effect of alternating fields. This is due to the fact that at high enough frequencies, there is not enough time for decomposition of water to form large hydrogen and oxygen bubbles that could affect the homogeneity of the coatings significantly. Bubble formation could lead to a change in porosity and roughness of the coatings. However, in this case, the changes are in such a small dimensional range, that there should be no influence on coating quality and adhesion to the substrate. Due to the electric field that leads to decomposition of water, the forces under AC fields are the same forces that drive the current through the double layer resistance. Thus, at high frequencies the current only flows through the double layer capacity and water is not decomposed or only a small amount of water molecules are dissociated, therefore there is no bubble formation [7]. Additionally, AC–EPD offers the possibility to deposit biological entities like cells or enzymes. It has been shown that after optimized AC–EPD conditions cells are still active and the functionality of enzymes or proteins remains [5]. Indeed, it is often not possible to deposit sensitive materials by DC–EPD as they are destroyed or inactivated during the process.

The use of natural polymers is of advantage to biomedical applications due to their excellent biocompatibility and biodegradability. Another advantage is that natural polymers are closer to the chemical composition of the extracellular matrix of biological systems (in comparison to synthetic polymers) and they can be degraded by enzymatic degradation [8]. Considering that codeposition of chitosan and biological species is relevant for a number of applications [8,9,10], there is interest in understanding the deposition of chitosan and chitosan based composite layers by AC–EPD. Chitosan is a natural polysaccharide [11], being a readily available, low-cost material. Chitosan is composed of glucosamine and *N*-acetyl glucosamine [12]. In comparison to other available biopolymers, chitosan has the ability to form films and chelate metal ions, which is not usually possible with other materials such as cellulose, pectin or agar [13]. Since chitosan is a suitable film-forming polymer, if deposited using EPD, no further heat-treatment is necessary [14]. Early reports about the electrophoretic deposition of chitosan are from 2002 [10]. Chitosan is positively charged and water soluble in acidic conditions at pH below 6.3. The positive charge results from the protonation of the amino groups [15].

The co-deposition of chitosan and bioactive glass (BG) has also been shown, mainly by applying DC–EPD [8,16]. As the deposition of pure BG coatings is not always of advantage due to the low mechanical strength and high brittleness of BG, the combination of BG with a polymer matrix to form composite coatings is of interest [17]. Indeed, given the superior osteoconductivity and bioactivity of BG [18], BG-chitosan coatings are attractive for orthopedic applications and bone tissue engineering because they will lead to superior bonding between the implant and the surrounding bone. Additionally such coatings are degradable at different rates, which makes them useful candidates for local drug or therapeutic ion release [9,19]. In particular, nanosized BG particles have been reported to show antimicrobial activity against various species [18].

In the present work, the electrophoretic co-deposition of chitosan-BG composite coatings with different BG particle sizes on different substrate materials was investigated. AC–EPD was used for the first time to produce this type of coatings aiming at reducing bubble formation and film disruption during the deposition process. The produced coatings were investigated according to their adhesion to the substrate, hydrophilicity and bioactivity. Antibacterial studies using *E. coli* were also conducted to provide a complete characterization of the produced coatings.

## 2. Results and Discussion

### 2.1. Deposition on Flat Surfaces

**Figure 1:**
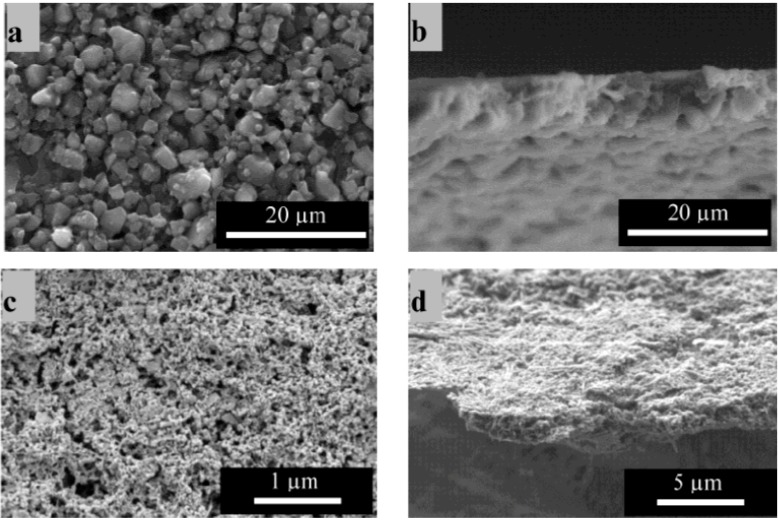
(**a**) Surface and (**b**) cross sectional views of chitosan-bioactive glass (BG) composites (containing BG micro particles); and (**c**) surface and (**d**) cross sectional views of chitosan-BG composites (containing nanoscale BG particles).

The preliminary EPD parameter optimization for chitosan-BG composite coatings was conducted using qualitative adhesion strength tests (Tape Test according to ISO 2409) and by visual inspection of the coatings. The parameters for deposition on planar stainless steel substrates were set at applied voltage of 40 V peak-to-peak, 2 kHz AC frequency and 4 min deposition time. For the microsized particles, a BG concentration of 1.6 g/L was found to be the optimum concentration to obtain homogeneous deposits, whereas for nanosized BG particles deposition could be only obtained for a particle concentration of 1.0 g/L. All other concentrations did not lead to a stable and homogeneous suspension or did not form deposits during the EPD process. The top view and the cross section of typical coatings can be seen in Figure 1. The coating thickness and the homogeneity of the microsized composite coatings are of high quality, according to scanning electron microscopy (SEM) images. Compared to equivalent results reported in the literature [8], the present coatings are thinner (around 3.5 µm), but seem to be more homogeneous and have a higher BG content. In addition, BG particles are seen to be well embedded and dispersed throughout the whole coating forming a compact composite structure. For the nanoparticle containing coatings, a fairly homogeneous distribution of the particles in the matrix could also be attained. The coating thickness was around 1.3 µm. Also in this case the BG particles were seen to be evenly spread throughout the whole coating. In both cases it is only possible to delaminate the coating from the substrate by strong bending, which means that the cohesion of the BG particles provided by the chitosan matrix is qualitatively high.

**Figure 2:**
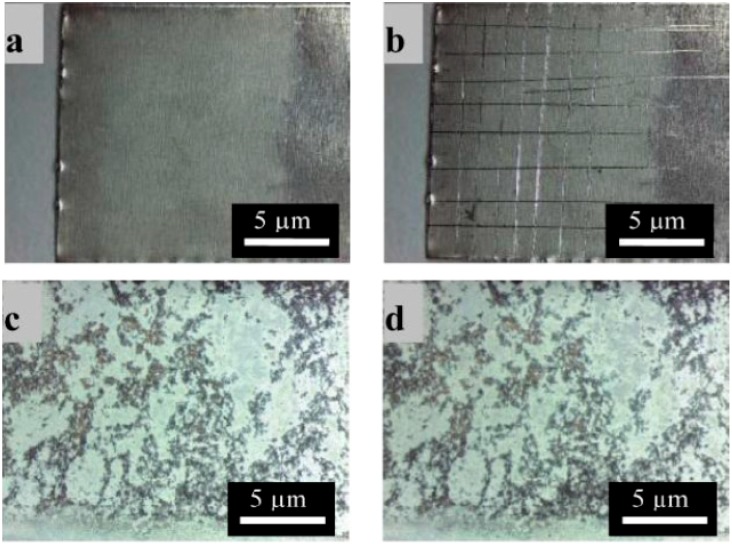
Optical microscopy images of chitosan micro BG coatings before (**a**) and after (**b**) Tape Test; and of chitosan nano BG coatings before (**c**) and after (**d**) Tape Test.

The adhesion of the coatings to the metallic substrate was investigated by applying the Tape Test. For the nano BG coatings, the Tape Test had to be modified. Normally coatings are cut into squares before the detachment operation, however this was difficult for the nano BG coating due to their strong adhesion to the substrate. No difference could be seen between cut and non-cut specimens, so only the non-cut results will be shown here. A strong adhesion of the coatings to the substrate is of interest as it is important for a safe handling of the product. Figure 2 shows the results of the Tape Test. For both coatings no differences before and after the Tape Test could be detected by visual inspection.

This observation leads to the conclusion that the adhesion of the coatings is qualitatively extremely good. Additionally, the low-magnification pictures show an overview of the macroscopic uniform character of the coatings. The microsized BG coatings are fairly homogeneous in thickness, whereas the nano BG coatings are rather inhomogeneous in their macroscopic morphology. This behavior can be explained by an agglomeration effect of the nanoparticles induced by the electric field, which has also been shown in literature by other authors [19]. When applying an electric field, the charge on the surface of the nanoparticles is reduced and particles are able to approach each other and agglomerate to form clusters of a few micrometers in size. This agglomerate formation leads to a rather inhomogeneous coating structure. The reduction of the applied voltage decreases this effect slightly, but small voltages also lead to very thin coatings or no coating formation. However, as the adhesion of the coatings is remarkably good, as mentioned above, the present chitosan-BG nanoparticle coatings represent promising surfaces for orthopedic applications and they were used for further experiments.

### 2.2. Deposition on 3D Structures (Scaffolds)

**Figure 3:**
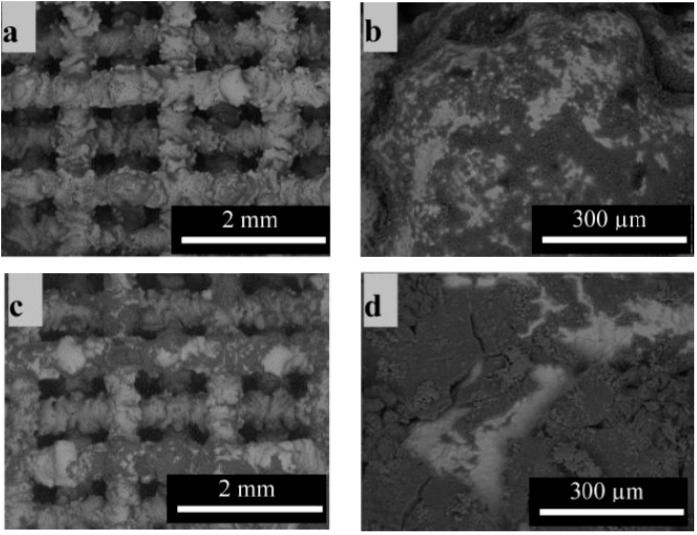
Scanning electron microscopy images of TiAl6V4 scaffolds with chitosan-BG composite coating containing (**a**) and (**b**) micro BG; and (**c**) and (**d**) nano BG particles.

For the deposition on 3D substrates, the EPD parameters had to be modified. The concentration of BG micro-particles was increased to 2.6 g/L, whereas the one for nanosized particles was kept as for 2D substrates. The deposition time was increased to 40 min. Preliminary trial-and-error experiments had shown that these parameters could lead to the best results in terms of coating homogeneity. SEM images of the coatings formed on the 3D structures are shown in Figure 3. The black areas correspond to the coating, whereas the light grey area is the substrate material, *i.e.*, complete coverage of the struts could not be attained. However, for the application of these 3D scaffolds in bone regeneration, a complete coverage is not strictly required because a rough surface could be more conducive to cell attachment. The scaffold images also show that the coating is not only present on the outside of the scaffold but also in the interior of it, which proves the adequate infiltration into the 3D structure using the optimized parameters.

### 2.3. Bioactivity Study in Simulated Body Fluid (SBF)

**Figure 4:**
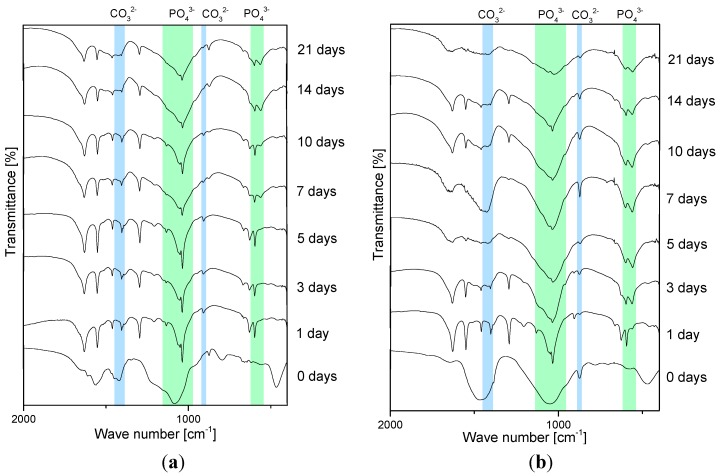
Fourier transform infrared (FTIR) spectra of chitosan/BG composites coatings. (**a**): microsized BG; and (**b**): nanosized BG.

After the successful deposition of the coatings, both 2D and 3D samples were immersed in SBF for several time periods of up to 21 days to investigate their bioactivity, which is determined by formation of hydroxyapatite (HA) on the surface [20]. Fourier transform infrared (FTIR) spectra of 2D coatings (Figure 4) show the changes in the composition of the coatings after several time periods in SBF. The characteristic peaks confirming HA formation are marked in the graphs. The most important peaks that occur during HA formation are discussed according to Stoch *et al*. [21]. For the evaluation of carbonated HA formation, phosphate and carbonate bonds are of interest. In particular, phosphate groups show four modes that are active in the infrared region: (i) bending vibration of PO_4_ at 560–610 and 430–460 cm^−1^; (ii) asymmetric stretching: broad band at 1000–1150 and at 960 cm^−1^.

It should be noted that carbonated HA has characteristic peaks for carbonate bonds as well. Those bonds are sometimes difficult to evaluate for composite materials with a polymeric matrix like chitosan as the polymer also has peaks in the same region, namely C-O vibrations at 1410–1470 and 850–890 cm^−1^. The observed differences in the HA formation between nanosized and microsized coatings are likely due to the fact that the surface area of the BG nanoparticles is much higher than that of microparticles which should enhance HA formation on the surface.

**Figure 5:**
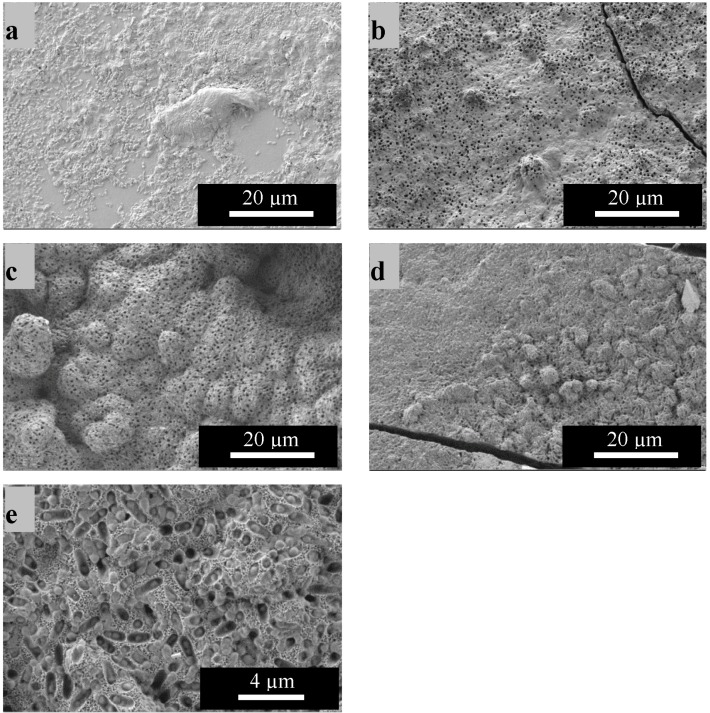
SEM images of chitosan-BG coatings after immersion for 21 days in simulated body fluid (SBF) for BG micro-particle composites on 2D (**a**) and 3D (**c**) substrates and for nanosized BG composites on 2D (**b**) and 3D (**d**,**e**) substrates.

Additionally, Figure 5a–e shows SEM micrographs of the samples immersed for 21 days in simulated body fluid (SBF). From the images it is apparent that when comparing the micro and nanosized BG particles on 2D substrates, coatings obtained from the nanosized particles show a higher HA forming ability as the HA coating is much thicker (Figure 5b). This result can be attributed to the higher surface area of the particles leading to a higher ion exchange with the surrounding medium resulting in accelerated HA formation. These results are in agreement with published studies, where higher bioactivity was found for BG nanocomposites in comparison to BG microcomposites [22].

On the 3D structures (Figure 5c,d) after 21 days of immersion, large areas of the surface are covered with the characteristic cauliflower like HA structure. The difference in morphology between the coatings of 2D and 3D microsized BG composites can be attributed to the higher BG amount in the suspension used for deposition on 3D substrates leading to higher BG contents in the coating A problem occurring when coating 2D substrates (as well as when using higher BG concentration) was that the coating showed propensity to microcracking which reduced the adhesion to the substrate. For the 3D structures the difference in the rate of bioactivity between micro and nano BG particle coatings is not significant. A clearer understanding of the evolution of surface reactions could be obtained using in situ measurements during HA formation, but also a time-dependent evaluation of cross sections of the formed coatings should be carried out. The images also show that there are many small pores in all HA layers. One possible explanation for this result could be the dissolution of chitosan from the layer. The BG particles in the coatings are surrounded by chitosan as a matrix material. During immersion in SBF, pores can evolve by the (partial) dissolution of the chitosan matrix. On the other hand, it is also likely that during HA formation, residual (partially reacted) BG particles are entrapped in the HA coating and with longer immersion time, these particles will dissolve, leaving residual porosity. This assumption is strengthened by considering the larger magnification image (Figure 5e).

### 2.4. Antibacterial Study

**Figure 6:**
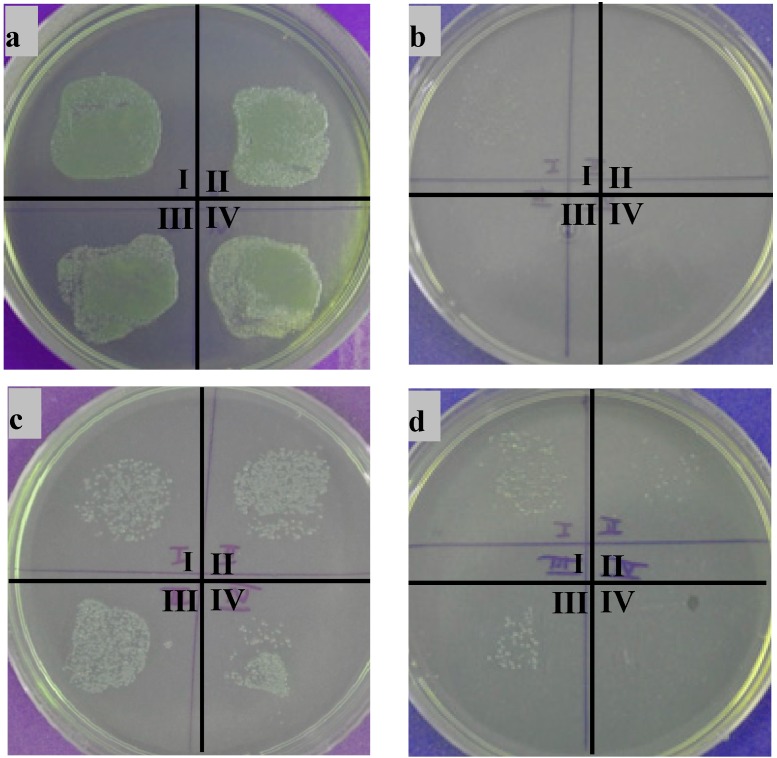
Results of antibacterial studies on different samples: (**a**) pure stainless steel; (**b**) chitosan; (**c**) chitosan/BG; and (**d**) chitosan/nano BG.

It has been reported that BG shows antibacterial behavior [23] and given that chitosan itself is antibacterial [24], a strong antibacterial effect of the composite coatings was expected. As reference samples, the bare stainless steel substrate and pure chitosan coatings were used. The different quarters in the images refer to the different immersion periods of 1 (I), 2 (II), 3 (III) and 4 (IV) h. It is obvious from the digital images in Figure 6a that steel does not inhibit bacteria growth. There are many bacteria colonies visible after all time periods. On the other hand, pure chitosan coatings showed a very good antibacterial behavior (Figure 6b). Unexpectedly the antibacterial effect of the composite coatings is not as strong as that of pure chitosan. This result can be explained by the fact that the coatings had different roughness characteristics, which gives a better chance for bacterial adhesion in the case of the composite coatings which exhibit higher degree of roughness. It is also possible that, due to swelling of the chitosan coatings, bacterial cells became entrapped in the coatings and were not stamped on the agar plate leading to an artificially high antibacterial effect of the pure chitosan coatings. Additionally, the antibacterial effect of BG, which is related to an increase of the pH of the surrounding medium by the BG dissolution products, may be limited in composite coatings because the area of BG particles directly exposed to the surface is rather small, which hinders large pH changes hence reducing the antibacterial effect in comparison to “pure” BG for the short incubation times investigated.

In comparison to coatings made from microsized BG, nanoBG coatings showed an increased antibacterial effect. The increased antibacterial properties of nanosized BG particles in comparison to micrometric ones have been highlighted in the literature [25]. This is the reason why nanoparticles containing coatings were investigated and in comparison to the micrometric particles a strong antibacterial effect was determined (Figure 6d). There are some bacterial colonies left after 1, 2 and 3 h, whereas after 4 h no colonies are visible, demonstrating the effective antibacterial behavior of the BG chitosan coatings enhanced by nanoscale BG. The slightly higher amount of bacteria after three hours in comparison to two hours can be explained by the manual stamping process. A slightly different stamping angle from sample to sample can lead to changes in the transfer of bacteria from the sample to the agar plate. Additionally, chitosan swells in contact with LB medium, which can lead to an entrapment of bacteria colonies that will not be transferred to the agar plate.

## 3. Experimental Section

### 3.1. Materials

For the electrophoretic deposition of chitosan (Sigma Aldrich, Schnelldorf, Germany), the polymer was dissolved in a mixture of deionized water and 1 vol % acetic acid following a previously published protocol [8]. The mixture was stirred for 24 h using a magnetic stirrer. Afterwards, bioactive glass (BG) particles of 45S5 composition (nominal composition in wt %: 45 SiO_2_, 24.5 Na_2_O, 24.5 CaO and 6 P_2_O_5_) were added to the suspensions, which were homogenized during 5 min in an ultrasonic bath followed by 15 min stirring. Two different BG particles with different particle sizes were investigated in this study, namely a commercially available BG powder with a mean particle size of 2 µm and a nanosized BG (mean particle size: 20–80 nm) produced using flame spray synthesis [26] (kindly provided by ETH Zurich, Zurich, Switzerland). As it was not possible to obtain a well dispersed suspension for nano-BG in pure aqueous suspensions, for these experiments a mixture of 75 vol % ethanol in water was used for EPD. For the nanosized particles a concentration of 1.0 g/L was used, whereas the concentration of the microsized particles was changed depending on the substrate material. Two different substrate materials were used. On the one hand, planar stainless steel 316 L (Advent Research Materials, Oxford, UK) foils with a thickness of 0.2 mm and a deposition area of 1.5 × 1.5 cm^2^ were used. On the other hand metal foams made from TiAl4V6 alloy produced using selective electron beam melting (produced by Chair of Metals Science and Technology, University Erlangen-Nuremberg, Erlangen, Germany) were used. Both materials are common materials for biomedical applications. Preliminary experiments showed that it is necessary to pretreat the titanium alloy to remove the oxide layer because this layer acts as an insulator preventing the successful deposition of the coating. A pretreatment in sulfuric acid for 24 h with subsequent rinsing in deionized water was shown to be the most promising method.

### 3.2. Electrophoretic Deposition

Different deposition parameters were investigated. The deposition parameters for the different substrates differ slightly due to the different conductivity of the substrate materials used. For all cases an electrode distance of 0.5 cm was chosen and the chitosan concentration was kept constant at 0.5 g/L. According to coating homogeneity and adhesion to the substrate, which was investigated by the Tape Test (according to ISO 2409) using an Elcometer 107 Cross Hatch Cutter (Elcometer Instruments GmbH, Aalen, Germany), the best parameter combinations were chosen for the planar substrates. The tested samples were checked using a Leica M50 optical microscope (Leica Microsystems GmbH, Wetzlar, Germany). For parameter optimization on 3D substrates, the homogeneity of the coatings was investigated by SEM Zeiss Auriga (Carl Zeiss Microscopy GmbH, Jena, Germany).

### 3.3. Characterization

The produced coatings were investigated according to their hydroxyapatite forming ability in simulated body fluid (SBF), which is the well-known test to assess bioactivity [20]. SBF is a solution with ion concentrations similar to that of human blood plasma. The SBF used in this project was prepared according to Kokubo *et al.* [20]. Samples with a size of 1.5 × 1.5 cm^2^ in cross section were immersed in 50 mL SBF for up to 21 days in an orbital shaker IKA KS 4000i control (IKA, Staufen, Germany). The samples were examined using an FTIR Nicolet spectrometer (Thermo Fisher Scientific Inc., Waltham, MA, USA,) and scanning electron microscopy (SEM) Zeiss Auriga (Carl Zeiss Microscopy GmbH, Jena, Germany).

The investigation of the antibacterial properties of the coatings was carried out using *E. coli* dH5α bacterial cells. The bacterial cells were incubated overnight and the solution was then diluted to an optical density OD600 of 0.01. As bacteria medium, LB Medium (Luria/Miller) (Carl Roth GmbH, Karlsruhe, Germany) was used. Exactly 20 µL of the suspension and 40 µL bare bacteria medium were placed on each sample using a pipette. The samples with bacteria drops were subsequently placed in an incubator to let the bacteria grow for 1, 2, 3 and 4 h. After each time period one sample was stamped on an agar plate (LB agar) and the agar plates were placed in an incubator to let the bacteria cells grow for 24 h. Digital camera images were then taken to assess bacterial growth.

## 4. Conclusions

Chitosan was used as a matrix material to embed bioactive glass particles from different particle sizes to form bioactive, antibacterial coatings by AC–EPD. Adhesion tests showed a qualitatively good adhesion of the produced coatings to the substrates and SEM images showed homogeneous coating formation on planar substrates. In the case of 3D TiAl4V6 alloy scaffolds, although the struts could not be fully covered, still a fairly homogeneous coating could be formed and the whole volume of the scaffold was coated. Studies in simulated body fluid confirmed the growth of hydroxyapatite on all coatings, demonstrating the bioactivity of the produced coatings. However, a difference between microsized and nanosized composite coatings was found, which can be explained by the BG particle size and the composition of the composite. Additionally, bactericidal studies were conducted. Bacterial studies using *E. coli* showed an antibacterial effect for both produced composite coatings on planar substrates. However, the antibacterial effect was significantly higher for nanosized BG particles in comparison to microparticles. Thus, this study showed that it is possible to produce robust bioactive and antibacterial chitosan-BG coatings by AC–EPD that have the potential for applications in orthopedics and bone tissue engineering. The specific effect of the nanosize *vs.* micronsized BG particles on the antibacterial effect and biological compatibility of the new coatings remains as an interesting subject for future research.
